# Shade Selection in Esthetic Dentistry: A Review

**DOI:** 10.7759/cureus.23331

**Published:** 2022-03-20

**Authors:** Mohammed Odhayd Alnusayri, Mohammed Ghazi Sghaireen, Merin Mathew, Bader Alzarea, Vinod Bandela

**Affiliations:** 1 Prosthetic Dental Sciences, College of Dentistry, Jouf University, Sakaka, SAU

**Keywords:** manual shade selection, spectrophotometer, shade selection, shade matching, aesthetics, shade guide

## Abstract

When fabricating a restoration, patient satisfaction with the shade match is essential. The patient's level of satisfaction may not be the same as that of the dental practitioner. Esthetic expectations have dramatically increased in the last few years. Color and shade determination are generally considered difficult in dental practice. Most prostheses fail esthetically owing to improper shade selection. A good understanding of color shade guides is essential for precise shade matching, but the paucity of dental practitioners’ knowledge of color science because of the numerous available shade guides creates a challenge. This comprehensive review sheds light on shade selection, different shade guides, and shade matching devices commonly used in dental practice. Studies published in PubMed/MEDLINE, ScienceDirect, Scopus, and Dentistry and Oral Science Source from the past 15 years were included.

## Introduction and background

Esthetics has gained wide importance over the last few years among both dental practitioners and patients. It is essential to deliver an esthetic restoration that symmetrically blends with the patient's adjacent teeth [1]. However, owing to the extensive range of natural tooth colors, achieving an intimate shade match between the prosthesis and natural teeth is challenging. Many prostheses fail due to improper shade selection, and therefore, every dentist should be acquainted with the shade selection process to achieve the best outcomes. The human brain can identify nearly one million shades, and precise devices that can recognize approximately 10 million different shades have been developed. Human dentition shades differ significantly, and electronic devices can identify approximately 100,000 dental shades while the human eye can identify only 1% of these shades [2].

It is difficult to accurately describe and verbally communicate shades; hence, three variables are used to characterize the perception of light reflected from the tooth surface: hue, value, and chroma. Hue describes the dominant shade of the tooth (more yellowish or reddish), value is the lightness or darkness of the tooth shade measured independently of the hue, and chroma is the quality that distinguishes the degree of vividness of the hue [3]. This review article includes studies on shade selection and shade matching devices used in dentistry. Studies published in PubMed/MEDLINE, ScienceDirect, Scopus, and Dentistry and Oral Science Source from the past 15 years were searched. Data extracted and the quality of studies assessed.

## Review

Guidelines for clinical shade selection

Shade-matching devices are also called shade guides. The order of shade selection is first selecting the value, chroma, and lastly hue. Color matching should be done in a systematic way that ensures accuracy, uniformity, predictable results which are absolutely important in esthetic dentistry.

Operating Site Lighting

Sunlight in the middle of the day is considered optimal for shade selection, as this exposure contains an almost equal blend of all wavelengths of light compared to morning and evening exposures, which are richer in reddish and yellow wavelengths. In clinics that do not have proper access to sunlight, artificial light should be used to simulate sunlight. Although no artificial light lamp can perfectly duplicate sunlight, it is adequate for clinical purposes. Prior to shade selection, the light to which the patients are most exposed to in their daily routine must be ascertained [1,3-5].

Environment

Bright-colored surroundings should be avoided as they interfere with proper color matching by influencing the colors in the reflected light. A drape can be used to mask undesirable colors in the patient's clothing and jewelry. Lipstick should be removed so that it does not affect color perception. A very light gray color provides the ideal background for color matching. Surfaces with high gloss produce disturbing glares and should be avoided [1,3-5].

Condition of the Teeth

The tooth of interest and its adjacent teeth should be free of plaque and other deposits and surface stains. The tooth should be moist with saliva as dehydration results in a whiter appearance. The tooth becomes dryer after application of the rubber dam, and therefore, color matching should be performed before applying it [1,3-5].

Distance of the Operator From the Tooth, Position of the Patient, and Timing

A distance of 61 cm (2 feet) to 183 cm (6 feet) distance from the oral cavity is considered ideal for shade matching [1,3-5]. The patient should be positioned in the dental chair such that the patient’s teeth are at the level of the operator's eyes. The operator should stand directly in front of the patient, with light focused on the teeth [1,3-5]. Shade selection and shade matching should be performed by the dentist preferably in the morning, when eye fatigue is minimal [1,3-5].

Squint Test for Restricting Light

The squint test enables shade selection by restricting the light entering the eye. It is performed by bringing the eyelids closer and then looking at the shade guide and the natural tooth. The color that fades from view first is the one that is least conspicuous in comparison with the tooth color [6]. There are two types of shade selection methods: the conventional method and the use of color measuring instruments [1].

Visual shade guides

The conventional method of shade selection is the use of visual shade guides which are the most famous and convenient way in selecting tooth shades. They are cost-effective and readily available; they also proficiently match the color of the dentition with a standardized reference shade guide. The selection of tooth color by the shade tab method completely depends on human eye observation. The currently available shade tabs are Vita classical (Bad Säckingen, Germany: VITA Zahnfabrik H. Rauter GmbH & Co.), Vita Toothguide 3D-Master shade guide (Bad Säckingen, Germany: VITA Zahnfabrik H. Rauter GmbH & Co.), and Chromascop (Buffalo, NY: Ivoclar Vivadent Inc.) [7].

Vita Classical Shade Guide

Based on the hue, 16 tabs are arranged into four groups and within the groups corresponding to the chroma. Since there are some limitations with Vita classical shade guide, Vita 3D-Master shade guide is the most commonly used among the commercially available shade tabs. It provides superior and standardized color differences [8,9].

Vita Toothguide 3D-Master

It comprises 26 tabs separated into five groups depending on the lightness of the color. The numbers (1, 2, 3, 4, and 5) in front of the letters represent the group number and lightness level; a lower number indicates a higher lightness. The numbers (1, 1.5, 2, 2.5, and 3) below the group number represent the level of the chroma; the more chromatic tabs are indicated by larger numbers. Three bleaching shades (0M1, 0M2, and 0M3) indicate more lightness, three levels of chroma, and middle hue. The major contrast between the Vita classical and Vita 3D-Master is that the Vita classical shade guide is built on the color hue and the Vita 3D-Master characterizes the color value. The Vita 3D-Master shade guide is considered superior to the Vita classical shade guide. It contains enhanced lightness spectrum and additional chromatic tabs. The hue latitude is expanded against the reddish spectra. Further, the shade tabs are evenly distributed and group division is improved [10-12].

Chromascop

Chromascop uses a numbering system to identify shades. It is organized into groups depending on the hue (100 = white, 200 = yellow, 300 = orange, 400 = gray, 500 = brown) and within the groups as chroma increases from 10 to 40 [3].

Custom Shade Guides

The standard shade guide cannot encompass the entire range of hue and chroma values of human dentition. It is useful for 85% of the color selection, and its alteration or preparation of custom shade tabs is necessary for the remaining 15%. Composite resin, ceramic, or acrylic materials are used to fabricate custom-made shade guides. Shade guide modifications can be performed using surface colorants or by surface abrasion using aluminum oxide. Fine line markers and colored pencils may be used to reproduce the minute variations between shades, analogous translucency, and denominating colors [13].

*Dentin and Extended​*​​​​​​* Shade Guides*

Dentin system can be used for the fabrication of translucent all-ceramic crowns and veneers. This shade guide helps in communicating a specific shade to the dental laboratory. Specially colored die materials corresponding to the dentin shade are used, which allows the technician to appraise the esthetics of the restoration [14]. The extended shade guide comprises the tabs of all materials used to fabricate the restoration. It may also be utilized to expand the choice of shade [15].

Disadvantages of shade guides

The disadvantages of shade guides are described as follows: (a) the colors in shade guides differ for each manufacturing company; (b) porcelain used for restoration may not be identical to that used in a guide; (c) guides are unable to direct the fabrication of porcelain restorations; (d) the shades in a guide are not logically organized and do not cover the volume of color space that exists in natural dentition; (e) a standard shade tab is made using synthetic resin and has greater thickness than that of a crown; (f) a shade guide tab reflects and transmits light, creating translucency and an appearance of vitality [16,17]. However, for a restoration, light is reflected and is less likely to be transmitted, which makes the restoration appear dense and opaque.

Color measuring instruments

All color-measuring devices comprise three parts: a detector, signal conditioner, and software that converts the signal into data that can be used in the dental laboratory or operatory. Following are examples of color measuring instruments: (1) colorimeters, (2) spectrophotometers, (3) digital cameras, (4) hybrid devices, and (5) spectroradiometers.

Colorimeter

A colorimeter measures color (hue, chroma, and value) as perceived by a human eye. It can only measure color by measuring tristimulus values under fixed illumination and observer conditions. The light source, integrating sphere, and detector (three or four filters) are the key optical elements [6,16].

Spectrophotometer

Spectrophotometers are commonly used to analyze surface colors. They measure the amount of spectral reflection from the body. It is a photometer that can measure intensity based on color, or more specifically, wavelength. The optical elements consist of a light source, monochromator, and detector. In general, light sources are diffracted. Several wavelengths are passed through the entrance slit and test sample to be tested [18]. Different wavelengths of light are selectively absorbed by the sample. The light then passes through another slit, called the exit slit, and strikes the detector. The detector converts the intensity of light at a certain wavelength into an electrical signal, which is then amplified and displayed on a screen or plotted on a chart. It is advisable to use a spectrophotometer to accurately measure color. A colorimeter provides an overall measure of the light absorbed, while a spectrophotometer measures the light absorbed at varying wavelengths. Briefly, colorimeters measure the amount of light absorbed overall, while spectrophotometers measure the amount of light absorbed by a specific wavelength. Spectrophotometers are reliable and accurate over time [19].

Digital Cameras

A digital camera is the most basic form of an electronic shade-matching device. In contrast to film cameras, this device records images using charge-coupled devices (CCDs), which comprise thousands or even millions of minute light-sensitive elements known as photosites. It provides a thorough and precise picture of the tooth surface and is also useful for color mapping. There is a flashcard that records all memories and allows the recording of voice feedback that can be sent directly to the lab without the need for a computer. The data can be downloaded onto a computer system for easy shade and translucency mapping [20,21].

Hybrid Devices

SpectroShade provides a combination of digital imaging and spectrophotometric analyses. It uses ClearMatch software system (Hood River, OR: Smart Technology) and is a hardware-independent product developed for use on all personal computers having the Windows platform and almost any digital camera [22].

Spectrophotometers and Spectroradiometers

These instruments allow the most precise color measurements. A spectrophotometer differs from a spectroradiometer in that it mainly contains a steady source of light. Two different basic designs have been employed for these instruments. The conventional scanning device comprises a single photodiode detector that records the quantity of light at each wavelength [23]. The latest design utilizes a diode array with a dedicated element for each wavelength. This design allows the simultaneous integration of all wavelengths at the same time. Both designs operate significantly slower than filter colorimeters but remain important for research on the development of precise color-measuring devices [24].

Limitations of digital shade guide

The limitations of digital shade guide include the following: (a) the phenomenon of edge loss affects the accuracy of color measurement; (b) translucent mapping is inadequate for all systems; (c) placement of the probe or mouthpiece seems to be important for the repeatability of the measurement; (d) no digital shade guide is sufficiently advanced to operate in a formulation mode; (e) the laboratory must have up-to-date systems for the successful application of this approach; (f) this approach requires a relatively expensive setup [21,25].

## Conclusions

Selection of the correct shade is the foundation for superior esthetics. Determining and closely reproducing the appearance and form of teeth is challenging. To provide an esthetic restoration to the patient, the dentist must have a full understanding of the science of color and color perception. Matching the correct shade satisfies both the dentist and patient and gives a pleasing appearance to the patient. In order to achieve good esthetics, both conventional and digital shade systems should be employed during the shade selection process.
